# Has the COVID-19 pandemic changed the utilization and provision of essential health care services from 2019 to 2020 in the primary health care network in Lebanon? Results from a nationwide representative cross-sectional survey

**DOI:** 10.1371/journal.pone.0288387

**Published:** 2023-07-13

**Authors:** Sally Yaacoub, Carla Zmeter, Linda Abou Abbas, Enrica Leresche, Ola Kdouh, Rawan Hammoud, Jennifer Leaning, Randa Hamadeh, Claudia Truppa

**Affiliations:** 1 International Committee of the Red Cross, Lebanon Delegation, Beirut, Lebanon; 2 Université Paris Cité and Université Sorbonne Paris Nord, Inserm, INRAE, Center for Research in Epidemiology and Statistics (CRESS), Paris, France; 3 London School of Hygiene and Tropical Medicine (LSHTM), London, United Kingdom; 4 Global Health Team of Experts, Ministry of Public Health Lebanon, Baabda, Lebanon; 5 Harvard FXB Center for Health and Human Rights, Boston, MA, United States of America; 6 CRIMEDIM—Center for Research and Training in Disaster Medicine, Humanitarian Aid and Global Health, Università del Piemonte Orientale, Novara, Italy; Cornell University College of Veterinary Medicine, UNITED STATES

## Abstract

There is limited research soliciting the patient and staff perspectives on the overall effects of COVID-19 on the utilization and provision of primary care in Lebanon. The present study was part of a larger study on the overall effect of COVID-19 on both utilization and provision of essential health care services within the Lebanese primary health care network (PHCN). Here, we present the patient and staff perspectives on continuity of service provision, adherence to infection prevention and control measures, and the role of the PHCN in epidemic preparedness and response. We conducted a cross-sectional survey between June and July 2021 among patients who had received a health care service in 2019 or 2020 from registered primary healthcare centers (PHCs) in the network and among the respective PHC staff working during the same period. A total of 763 patients and 198 staff completed the surveys. Services were reported as interrupted by 15% of the total patients who used services either in 2020 only or in both 2019 and 2020. Access to chronic (67%) and acute medications (40%) were reported as the main interrupted services. Immunization also emerged as a foregone service in 2020. Among the staff, one third (33%) reported interruptions in the provision of services. Financial barriers rather than fear of COVID-19 were reported as main reasons for interruption. Both groups considered that the facilities implemented adequate infection prevention and control measures. They perceived that the PHCN maintained some essential healthcare services and that it should have played a bigger role in the response to the pandemic. There was a continuity in utilization and provision of services in the PHCN that was higher than expected, with non-communicable diseases and immunizations suffering more than other services.

## Introduction

The COVID-19 pandemic has put a significant strain on national public health systems around the world. As countries scrambled to respond to the crisis, many were forced to divert resources and personnel away from routine health care services in order to address the immediate needs of COVID-19 patients [1]. These disruptions have been particularly severe in low and lower-middle income countries (LMICs), where pre-existing public health infrastructure is already weak [2, 3].

The World Health Organization (WHO) conducted a global pulse survey on the continuity of essential health services during the COVID-19 pandemic and found that more than 90% of surveyed countries reported disruptions in at least one essential health service, with the most affected services being immunization and non-communicable diseases (NCD) care [3]. Despite the efforts to maintain continuity of service provision, there has been no noticeable improvement in the magnitude and extent of disruption since early 2021, with high-income countries reporting less disruption than LMICs [2]. This decline is attributed to service and supply chain disruptions, resource allocation, containment measures, an increase in misleading information, and the increase in the number of children living in unstable and conflict-ridden areas where getting immunizations is frequently difficult [4]. There are also concerns about a potential increase in maternal and child deaths in LMICs as a result of the pandemic’s indirect effects [5].

As a result of its prolonged nature and the findings highlighted above, there is an increasing concern that the COVID-19 pandemic will have extensive indirect consequences on health outcomes, particularly in LMICs. This concern is attributed to the vulnerability of the pre-existing poor public health infrastructure combined with the diversion of necessary medical resources to the provision of COVID-19 cases prevention and care [5–7]. Sustained decline in health care utilization could reverse years of advancement in improving health outcomes in LMICs, which has been driven in large share by provision of essential services through primary health care (PHC) [8].

LMICs in the Middle East are particularly affected by conflict and fragility, which might exacerbate the impact of the pandemic on the provision of essential health care services. However, the only evidence in this area comes from upper middle- and high-income countries exclusively. For example, one study from Iran investigating the impact of the COVID-19 pandemic on the utilization of PHC services revealed a reduction in the number of essential services provided by different categories of health care workers [9]. Another retrospective analysis conducted in Qatar to measure the impact of COVID-19 on preventive care showed a sharp decline in the utilization of the services to almost 40% reduction in May 2020 [10].

Over the past 40 years, PHC has played a significant role in improving health and well-being, resulting in decreases in maternal, neonatal, and child mortality, as well as deaths from diseases such as HIV/AIDS, malaria, tuberculosis, and vaccine-preventable diseases [11]. PHC has proven to be an effective and efficient way to address the primary causes of, and risk factors for, poor health, as well as to handle emerging health challenges. It has also been shown to contribute to reduce the total health care costs by placing an emphasis on promotion and prevention, providing early diagnosis and treatment for a variety of conditions, offering people-centered care, and reducing unnecessary hospital admissions [12–14]. There is now strong evidence demonstrating the effectiveness of the implementation of a comprehensive PHC approach, especially in LMICs, in ensuring equitable access to care and protection from financial hardship [8, 12]. Thus, PHC is widely acknowledged as a crucial pillar for achieving universal health coverage (UHC) leaving “no one behind” [8].

Lebanon’s health care system is characterized by a mix of public and private providers. The Ministry of Public Health (MoPH) oversees and regulates the public health care facilities, while private hospitals and clinics cater to those who can afford higher costs. The National Social Security Fund (NSSF) provides mandatory health insurance coverage for formal sector employees, and out-of-pocket payments remain a significant component of health care financing [15]. The Lebanese Primary Health Care Network (PHCN) has struggled to survive and evolve in the wake of sequential and intertwined crises which have include historical political compromises, the influx of Syrian refugees in 2011, the accelerating economic breakdown, and the blasts that hit Beirut on August 4, 2020 [16–18]. Despite the challenges faced, the PHCN has made substantial progress towards UHC, through defining a bundle of essential health care services and making it accessible with subsidized packages for all populations residing in the Lebanese territory [16, 17]. However, the compounded shocks that have hit the country have undermined the health system capacity to maintain essential health care services for an increasing number of vulnerable populations. These include Syrian refugees who are granted access to the same channels of health care as Lebanese through a network of PHC services embedded in the complex privatized system [17].

Assessing the effects of socio-political events, existing economic vulnerabilities, and the COVID-19 spread on the provision and utilization of health care within the PHCN is crucial to enable planning of services, especially in resource-limited countries such as Lebanon. A secondary analysis of routine monitoring data from the Lebanese Ministry of Public Health (MOPH) Primary Health Care Network Information and Communication System (PHENICS) between January 2019 and December 2020 revealed that the socio-political turmoil and lockdowns were the main factors preventing the use of PHC services in Lebanon, and that the patterns of utilization decline in 2020 did not match the geographic spread of COVID-19 in the country [personal communication from MoPH]. A study conducted in April 2020 found that the utilization of immunization services was reduced by 31% at the national level, with a greater reduction in the private sector [10]. These declines in immunization rates raise the likelihood of resurgence of vaccine-preventable diseases outbreaks and possible imposition of further burdens on the Lebanese health system already exhausted by the COVID-19 pandemic and now facing a cholera outbreak [18, 19].

The available evidence in the published literature is mainly focused on the analysis of primary or secondary utilization data collected from health facilities and the MoPH, while there is limited evidence examining the perspective of providers and patients as well as public health priorities of local populations. Evaluating their perceptions can provide important holistic information and can complement the available data on the overall effects of COVID-19 on both utilization and provision of essential health care services.

The present study was part of a larger study on the overall effect of COVID-19 on utilization and provision of essential health care services within the Lebanese PHCN, which, in this part, aimed to explore the patient’s and staff’s perspectives. The specific objectives were to:

1. Describe the changes in utilization of essential health care services in the PHCN from the patients’ perspective and in provision of these essential health care services from the staff perspective.
2. Identify reasons for any described change in both utilization and provision of services, from the perspective of the patients and the PHC staff, respectively.
3. Document the perception of the implementation of infection prevention and control (IPC) measures within the PHCN from the perspective of both patients and PHC staff, within the dimensions of service coordination, workforce, and logistics.
4. Understand the perceptions from both the perspective of the patients and that of the staff on the role of the PHCN in Lebanon in prevention of, preparedness for, response to, and recovery from health crises.

## Methods

### Study design and population

This study was a cross-sectional survey using pilot-tested questionnaires conducted over the phone by trained volunteers. We included those PHCs registered in the MoPH network in both years (2019 and 2020) that had also agreed to share their data with a third party. We included all patients or patient caregivers who were at least 18 years old and who had received a health care service in 2019 or 2020 from one of the sampled PHCs. We excluded patients with no available contact information.

The full list of staff working in the PHCN was provided by the MoPH. From that list, we included employees who were clinical staff providing the basic PHC services as per the national PHC standards (general physician/family physician, pediatrician, gynecologist, cardiologist/endocrinologist, dentist, and nurses) or administrative/managerial staff. We included only employees who were employed in the sampled PHC in both years (2019 and 2020) in order to describe the potential changes in the provision of essential health care services in the PHCN, taking the year 2019 as a reference. We excluded staff who worked in the PHC in only one of the years (2019 or 2020).

### Sampling

We followed a two-stage cluster sampling method. We first selected PHCs in the PHCN (i.e., clusters); and then selected staff and patients from each of the included PHCs. To select the PHCs, we used systematic probability proportional to size sampling relying on the number of consultations in each PHC in 2019 as the size measure. The sample size was 96 PHCs (out of a total of 175 PHCs) for a 95% confidence interval (CI), assuming a conservative 50% expected proportion of PHCs affected by services disruption and 10% precision.

For the patients, we estimated the sample size to be 768 patients based on a 95% CI, an estimated conservative proportion of 50% experiencing service change, with a precision of 5% and accounting for the cluster effect. Using simple randomization and accounting for a 30% expected response rate (based on the pilot and unpublished surveys by the PHC team in the MoPH), we selected 2592 patients from the patient lists for each PHC (27 patients per PHC).

For the staff, we estimated the sample size to be 192 staff taking into account an expected conservative proportion of 50% of staff who would perceive that the utilization of services was affected, with 95% CI and 10% precision. Using simple randomization and accounting for an expected response rate of 35% (based on the pilot and unpublished surveys by the PHC team in the MoPH), we selected 576 staff from the list of staff (6 staff per PHC).

We calculated the needed sample sizes using Epi Info version 7.1.2.0 (U.S. Center for Diseases Control and Prevention). The sample selection of the PHCs, patients, and staff was performed by the MoPH information technology (IT) administrator with the guidance of the research team.

### Data collection

The data were collected using pilot-tested questionnaires. The questionnaires were developed for the purpose of this study first in English, then translated into Arabic, and then back translated into English. They were administered to the interviewees (patients and staff) in Arabic.

The patient questionnaire included questions on socio-demographic characteristics; utilization of services; adoption of COVID-19 infection prevention and control measures in the PHC; and the perceived role of PHCs in response to COVID-19. The staff questionnaire included questions on socio-demographic characteristics; difficulties encountered with service continuity; measures adopted in the PHC to protect the staff, patients, and the community against COVID-19; and the perceived role of PHCs in the pandemic.

The data collection was conducted from June to July 2021 by Lebanese Red Cross volunteers. The teams underwent one-day training on research ethics (delivered by the first, fourth and seventh authors of this study) focusing on obtaining informed oral consent and on administering the questionnaires. The questionnaires were administered over the phone and the answers were directly recorded into Survey123 (ArcGIS, Esri, United States of America). The teams were supervised and supported by one or more staff from the International Committee of the Red Cross (ICRC) to address any inquiry or issue.

### Ethical considerations

This research project was reviewed and approved by the ethical review board of London School of Hygiene and Tropical Medicine (Ref: 22760), by the International Committee of the Red Cross (ICRC) Health Unit in Geneva, and by an independent group of experts within the Lebanese Ministry of Public Health.

Prior to participating in the study, oral consent forms were obtained from all participants. The consent process involved providing detailed information about the study’s objectives, procedures, potential risks and benefits, and the rights of the participants. Participants were given the opportunity to ask questions and clarify any concerns before providing their oral consent. It was explicitly communicated to the participants that their data would be treated with strict confidentiality, and they were informed of their right to withdraw from the study at any time without any repercussions.

This study adhered to the principles of research ethics, including the respect for autonomy, confidentiality, and the protection of participants’ rights. The study protocol complied with the guidelines outlined in the Declaration of Helsinki for research involving human subjects.

### Data analysis

We reported proportions and percentages for categorical variables (e.g. gender, nationality) and means ± standard deviations (SDs) for continuous variables (e.g. age, hours in PHC per week). We used the Chi-square test, Fisher’s exact test and the student’s t-test when applicable. We conducted multivariable analysis using the Generalized Estimating Equations (GEE) appropriate for correlated data and stated objectives. We identified the factors associated with the dependent variables: change in utilization of services from the patients’ perspective and change in provision of services from the staff’s perspective using ‘no change’ as the reference category. We included the characteristics of the patients and those of the staff (e.g., age, gender, and nationality) as independent factors. We reported crude and adjusted odds ratios (ORs) with 95% confidence intervals (CIs) and set a p-value < 0.05 for statistical significance. We used IBM SPSS Statistics for Windows, Version 26.0 (IBM Corp., Armonk, NY, USA) for statistical analysis.

## Results

### Description of the included population

**Fig 1:**
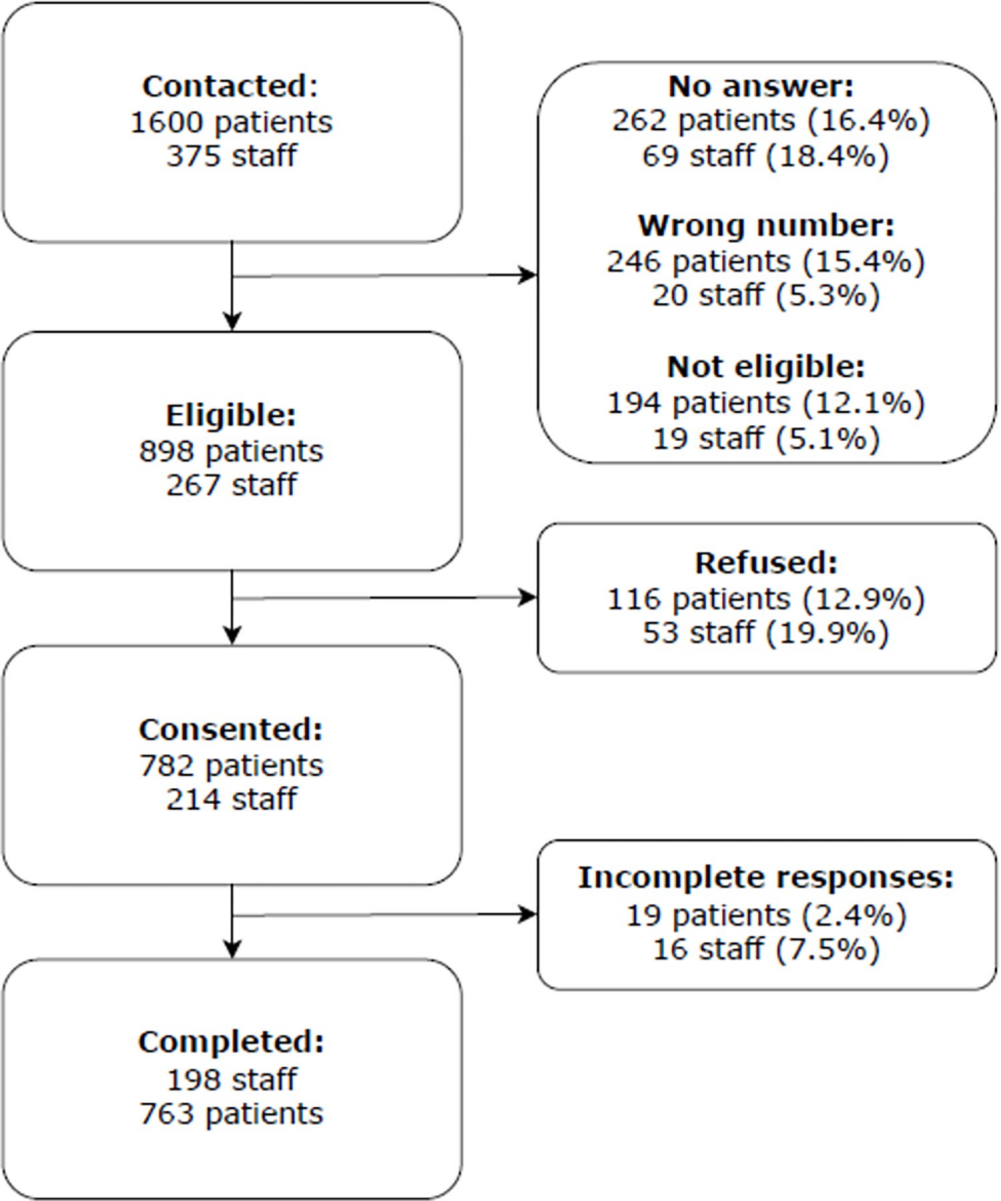
Flowchart of the patient and staff populations included in the study.

We contacted 1600 patients and 375 staff distributed across 96 PHCs in Lebanon (Supplementary material 1 in S1 File). Out of those contacted, 508 patients and 89 staff (32% and 24%, respectively) were not reachable, and once contacted, 194 patients and 19 staff (12% and 5%, respectively) were not eligible. From those who were reachable and eligible, 782 patients (87%) and 214 staff (80%) consented to be surveyed. Consequently, the response rate was 49% (n = 782, N = 1600) for patients and 57% (n = 214, N = 375) for staff. We excluded 19 patients and 16 staff due to incomplete responses, resulting in a total of 763 patients and 198 staff completing the surveys and their data analyzed. There was no statistical difference between the total sample and the participants reached in terms of age, gender or nationality. Additional details can be found in Fig 1.

### Patients

The mean age of these 763 patients was 40 years (SD = 12.4) and more than half were female (n = 425, 57%). Most of the patients were Lebanese (63%), with the non-Lebanese patients (37%) mainly including Syrian patients (98%, n = 277). More than half of the included patients were caregivers (56%). Of the total population surveyed, the caregivers were in general caring for children (80%) rather than elderly. The patients attended mainly PHCs in Mount Lebanon (42%) and attended for both years 2019 and 2020 (66%). More than half of the patients (58%) and 28% of primary earners were unemployed. Additional details on patient characteristics are presented in Table 1. Statistically significant differences were seen between the socio-demographic profiles of Lebanese and non-Lebanese patients (See Supplementary material 2 in S1 File).

**Table 1:** Characteristics of the patients included (N = 763).

Characteristic		Frequency	Percentage (%)
**Age** (Mean, SD)		40.0, 12.4	
**Gender** (N = 752)			
	Male	327	43,5
	Female	425	56,5
**Nationality** (N = 758)			
	Lebanese	475	62,7
	Non-Lebanese*	283	37,3
**Beneficiary** (N = 758)			
	Primary beneficiary	332	43,8
	Caregiver:	426	56,2
	for child	341	80,0
	for adult	73	17,1
	Unknown	12	2,8
**Governorate of PHC** (N = 763)			
	Mount Lebanon	323	42,3
	North	192	25,2
	Akkar	136	17,8
	Beirut	56	7,3
	Bekaa	16	2,1
	El Nabatieh	16	2,1
	South	16	2,1
	Baalbak El Hermel	8	1,0
**Year of attending the PHC** (N = 763)			
	2019 only	117	15,3
	2020 only	145	19,0
	2019 and 2020	501	65,7
**Employment status of beneficiary** (N = 756)			
	Unemployed	439	58,1
	Employed	317	41,9
	Full-time	122	38,5
	Part time	101	31,9
	Seasonal	73	23,0
	Self-employed	21	6,6
**Occupation of employed beneficiary** (N = 317)			
	Private sector	111	35,0
	Public sector	32	10,1
	Daily worker	156	49,2
	Other	16	5,0
	Not specified	2	0,6
**Employment status of breadwinner** (N = 745)			
	Not applicable	79	10,7
	Unemployed	207	28,2
	Employed	459	62,4
	Full-time	194	26,4
	Part time	137	18,6
	Seasonal	101	13,7
	Other	27	3,7
**Occupation of employed breadwinner** (N = 459)			
	Public sector	44	9,6
	Private sector	145	31,6
	Daily worker	216	47,1
	Other	49	10,7
	Not specified	5	1,1
**Education of beneficiary** (N = 749)			
	No education	144	19,2
	Elementary school	321	42,9
	High school	158	21,1
	University	126	16,8

SD: standard deviation

* Syrian (n = 277), Palestinian (n = 2), Sudanese (n = 2), Iraqi (n = 1), Armenian (n = 1)

### Staff

Among the total of 198 staff, the mean age was 40 years (SD = 12.6) and more than half were female (54%). Almost all the staff were Lebanese (99%) and worked in PHCs across Lebanon, mostly in Mount Lebanon (41%). The mean number of working hours in the PHC was 22.8 hours per week (SD = 18.5). Staff represented different occupations within PHC, including but not limited to nursing (26%) and general practice/family medicine (22%). Additional details on staff characteristics are presented in Table 2.

**Table 2:** Characteristics of the included staff (N = 198).

Characteristics		Frequency	Percentage (%)
**Age** (Mean, SD)*		44.2, 12.6	
**Gender** (N = 198)			
	Male	91	46,0
	Female	107	54,0
**Nationality** †			
	Lebanese	195	99,0
	Non-Lebanese	2	1,0
**Governorate**			
	Mount Lebanon	82	41,4
	North	49	24,7
	Akkar	34	17,2
	Beirut	14	7,1
	Bekaa	4	2,0
	El Nabatieh	9	4,5
	South	4	2,0
	Baalbak El Hermel	2	1,0
**Hours in PHC per week** (Mean, SD) **‡**		22.8, 18.5	
**Occupation**			
	General practitioner /Family physician	43	21,7
	Pediatrician	28	14,1
	Gynecologist	29	14,6
	Cardiologist	7	3,5
	Endocrinologist	5	2,5
	Dentist	21	10,6
	Nurse	52	26,3
	Administrative staff	13	6,6

SD: standard deviation

*N = 180

†N = 197; Non-lebanese include one Russian and one Palestinian staff.

‡N = 185

### Reported changes in utilization and provision of essential health care services

#### Patients

The majority of the patients interviewed attended the PHCN in both years 2019 and 2020 (n = 501, 66%) and the remaining attended the PHCN in one year only, either 2019 (n = 117, 15%) or 2020 (n = 145, 19%). There was no statistically significant difference between Lebanese and non-Lebanese patients in the proportion of patients attending the PHCN in 2019, 2020 or both years (crude OR = 1.606, 95% CI [0.960, 2.688]). Of the patients who attended the PHCN only in the year 2019, almost one third declared during the interview they would have needed to (but did not) seek multiple services from the PHCN in 2020 (n = 29, 28%), with a higher proportion of non-Lebanese (n = 14, 44%) compared to Lebanese patients (n = 15, 21%) (crude OR = 2.852, 95% CI [1.157, 7.029]). The most needed service would have been immunization (n = 17, 59%) (Supplementary material 3 in S1 File).

Interrupted services were reported by 15% of all patients (94 out of 635) who used services in 2020 (either in 2020 only or in both 2019 and 2020). Out of these 94 patients, 73 (78%) specified which services were interrupted. The main services that were interrupted were access to chronic (n = 49, 67%) and acute (n = 29, 40%) medication. Specifically, the disrupted access to chronic medications was considered as the ‘most important interrupted service’ by 65% (n = 47/73) of the respondents who specified the interrupted service. The PHCs most affected by service interruptions were in Akkar (n = 31, 33%), North Lebanon (n = 28, 29%) and Mount Lebanon (n = 23, 25%) (p-value = 0.001).

The multivariable analysis included a total of 548 patients (Table 3). Older patients had higher odds of having interruption of services (adjusted OR = 1.044, 95% CI [1.019, 1.069]). Patients of PHCs from Akkar governorate had higher odds of interruptions compared to those from Beirut governorate (adjusted OR = 4.831, 95% CI [1.353, 17.246]), while patients with high school degrees had higher odds of interruptions compared to those with no education (adjusted OR = 2.829, 95% CI [1.188, 6.735],). No other statistically significant factors were identified.

**Table 3:** Factors associated with the interruption of services from the patients’ perspective.

Characteristic		Adjusted odds ratio	Confidence Interval	P—value
**Age**		1.044	[1.019, 1.069]	**<0.001**
**Gender **				
** **	Male	1		
** **	Female	0.843	[0.463, 1.536]	0.577
**Nationality **				
** **	Lebanese	1		
** **	Non-Lebanese	1.650	[0.946, 2.880]	0.078
**Beneficiary**				
** **	Primary beneficiary	1		
** **	Caregiver	0.882	[0.553, 1.409]	0.600
**Governorate of PHC **				
** **	Beirut	1		
	Mount Lebanon	0.945	[0.301, 2.961]	0.922
	North	2.352	[0.705, 7.848]	0.164
	Akkar	4.831	[1.353, 17.246]	**0.015**
	Bekaa	4.680	[0.729, 30.025]	0.104
	El Nabatieh	1.914	[0.239, 15.341]	0.541
** **	South	0.694	[0.199, 2.420]	0.566
**Year of attending the PHC**				
** **	2020 only	1		
** **	2019 and 2020	0.654	[0.366, 1.169]	0.152
**Employment status of beneficiary **				
** **	Unemployed	1		
** **	Employed	0.764	[0.404, 1.447]	0.407
**Employment status of breadwinner**				
** **	Unemployed	1.000		
** **	Employed	0.791	[0.442, 1.416]	0.430
**Education of beneficiary**				
** **	No education	1.000		
** **	Elementary school	2.193	[0.964, 4.991]	0.061
** **	High school	2.829	[1.188, 6.735]	**0.019**
** **	University	2.725	[0.932, 7.969]	0.067

#### Staff

One third of the staff reported interruptions in the provision of services (n = 64, 33%). The multivariable analysis included a total of 174 staff (Table 4). Having a gynecologist present was protective against interruption of services (adjusted OR = 0.074, 95% CI [0.01, 0.560]); this was the only statistically significant factor associated with service interruption.

**Table 4:** Factors associated with the interruption of services from the staff’s perspective.

Characteristic		Adjusted odds ratio	Confidence Interval	P—value
**Age**		0.983	[0.95, 1.018]	0.341
**Gender **				
** **	Male	1		
** **	Female	1.972	[0.778, 4.997]	0.152
**Governorate**				
	Beirut	1		
	Mount Lebanon	1.824	[0.336, 9.917]	0.486
	North	2.058	[0.362, 11.694]	0.416
	Akkar	1.661	[0.264, 10.452]	0.589
	El Nabatieh	6.025	[0.599, 60.550]	0.127
	Baalbak El Hermel, Bekaa or South			
**Hours in PHC per week**		0.993	[0.965, 1.022]	0.638
**Occupation**				
	General practitioner /Family physician	1		
** **	Pediatrician	1.393	[0.433, 4.483]	0.578
** **	Gynecologist	0.074	[0.010, 0.560]	**0.012**
** **	Cardiologist	1.665	[0.178, 15.606]	0.655
** **	Endocrinologist	0.616	[0.103, 3.694]	0.596
** **	Dentist	0.461	[0.111, 1.921]	0.288
** **	Nurse	1.336	[0.380, 4.694]	0.651
** **	Administrative staff	0.647	[0.099, 4.234]	0.649

### Reasons reported for changes in utilization and provision of essential health care services

#### Patients

There were several reasons noted by new patients who began to attend the PHCN in 2020. These reasons were financial hardship (n = 107, 85%), affordability of the services in the PHC (n = 96, 81%), increased trust in the PHCN (n = 92, 78%), recent knowledge of the available services (n = 63, 53%) and presence of a specific doctor (n = 60, 52%). The proportion of new patients who started attending the PHCN due to financial hardships was statistically higher among non-Lebanese patients (n = 50, 96%) compared to Lebanese patients (n = 56, 77%) (crude OR = 7.589, 95% CI [1.670, 34.492]. Proportion attending the PHCN due to the presence of a specific doctor was lower among non-Lebanese patients compared to Lebanese patients (non-Lebanese: 40%, Lebanese: 61%) (crude OR = 0.426, 95% CI [0.199, 0.911]). Supplementary material 4 in S1 File presents the reasons reported by patients for not seeking the services that they needed from the PHCN in 2020.

For all the services interrupted, the reasons for interruptions were mainly financial barriers (n = 75, 82%), lockdown measures (n = 58, 68%), geographic barriers (n = 51, 59%) and fear of COVID-19 (n = 50, 56%) (Supplementary material 5 in S1 File). There were no statistically significant differences between Lebanese and non-Lebanese reporting the proportion of interrupted services or reasons for interruptions.

It is noteworthy that 34 patients reported receiving the needed services elsewhere, including at pharmacies (n = 15, 44%) and at another PHCC (n = 11, 32%).

#### Staff

The general reasons for interruption in provision of services reported by staff included financial concerns such as salary cuts/delays (n = 47, 75%); lockdown measures (n = 47, 75%); medication stockout (n = 44, 72%); and physical access issues, such as roadblocks, distance, and cost of fuel (n = 44, 70%). Vaccine stockout was also one of the reasons for service interruption (n = 28, 45%). The staff also reported facing personal constraints or difficulties in providing services. These constraints included concern about contracting COVID-19 (n = 137, 71%) or experiencing unsatisfactory financial arrangements (n = 132, 68%) (Supplementary material 6 in S1 File).

### Perception on implementation of measures for infection prevention and control

#### Patients

There were no statistically significant differences in the proportion of patients attending the PHC during the COVID-19 pandemic among Lebanese patients (n = 227, 59%) compared to non-Lebanese patients (n = 132, 54%) (crude OR = 0.843, 95% CI [0.610, 1.165]). Of all patients attending the PHC in 2020 (n = 646), more than half attended at the outset or during the COVID-19 pandemic (n = 360, 56%). Patients using PHC services during COVID-19 noted patients wearing masks (n = 357, 99%); staff wearing masks (n = 354, 99%); physical distancing (n = 338, 95%); and temperature checks (n = 332, 93%) as protective measures undertaken. Provision of educational information was the least reported measure (n = 253, 70%). Details on the measures undertaken in the PHC as reported by the patients are presented in Supplementary material 7 in S1 File. Overall, patients were comfortable in regards to COVID-19 while attending the PHC during the pandemic, with a mean of 8.5 (SD = 1.64) on a scale of 0 to 10 (10 being most comfortable).

#### Staff

The staff reported several measures that were taken during the pandemic to protect themselves, patients, and community (Supplementary material 8 in S1 File). One of the measures to protect the staff was having trainings on using the personal protective equipment (PPE) (n = 134, 68%). In general, the staff did not report having any difficulties protecting themselves (n = 122, 63%). The measures to protect the patients included temperature check (n = 187, 97%), use of PPE by staff (n = 177, 93%) and patients wearing masks (n = 178, 92%). To protect the community, the staff reported involving communities in the dissemination of COVID-19 awareness messages and dealing with rumors and misinformation (Supplementary material 8 in S1 File). There were also actions taken when a COVID-19 case was suspected. Out of the 79 (42%) staff who identified a suspected case presenting to the PHC in the previous month, 49 notified the MoPH through the hotline (65%). However, almost 50% of the staff (n = 39) had not personally used the MoPH hotline to report a suspected COVID-19 case identified in the PHC in the previous month, while in the majority of cases they either referred the patient to a hospital or laboratory, or instructed her/him to self-isolate (87% and 96% of cases, respectively).

### Perceptions related to the role of the PHCN during the COVID-19 pandemic

#### Patients

At the time of data collection, one quarter of the patients or their family members had contracted COVID-19 (n = 193) (non-Lebanese: 12.4%, Lebanese: 33.5%, crude OR = 0.281, 95% CI [0.188, 0.420]).

Among all respondents (including those who did not contract COVID-19), 43% of the patients would reach out to PHCs (n = 325, 43%) in case they or their family members contracted COVID-19. Other health facilities they would reach out to were governmental hospitals (n = 155, 20%), private hospitals (n = 75, 10%) and private clinics (n = 44, 6%). It is noted that non-Lebanese patients (n = 147, 56%) were more likely to express intention to reach out to the PHCs than Lebanese patients (n = 176, 40%) (crude OR = 1.934, 95% CI [1.418, 2.638]).

The reasons why patients would reach out to PHCs were mainly affordability of services (n = 157, 48%) and proximity of the PHC (n = 111, 34%). On the other hand, the main reasons why patients did/would not reach out to PHCs were lack of awareness of available services (n = 97, 26%), perceived lack of available testing modalities (n = 92, 24%), and availability of other health facilities (n = 66, 17%). There were no statistically significant differences between Lebanese and non-Lebanese patients in terms of reasons for reaching out the PHCs.

Patients benefitting from PHC services (n = 760) reported that it was important for them to have PHCs open and providing services with a mean of 8.5 (SD = 1.84) on a scale of 0 to 10 (10 being most important). Centers being open and providing services were more important concerns for Lebanese patients (8.7 ± 1.80) compared to non-Lebanese (8.3 ± 1.89, p-value = 0.004). Despite the perceived importance of keeping the PHCs open and providing services, respondents noted several areas for improvement—such as the availability of free medications (91%) and availability of a variety of medications (90%) (Supplementary material 9 in S1 File).

#### Staff

Of the 194 respondents, 67% thought that the PHC should have played a greater role in responding to the pandemic. This view was related to the belief that the PHC was more aware of people’s needs (n = 122, 95%); was closer to the communities (to relay health information and to provide medical support in a timely manner) (n = 120, 94%); and was better equipped to deal with issues related to health prevention and health behavior change (n = 103, 83%). The expanded role of PHCs suggested by staff included improved efforts to raise awareness (n = 126, 97%); to make referrals to health facilities (n = 124, 96%); to expand COVID-19 testing (n = 119, 92%); and to promote COVID-19 vaccination (n = 117, 91%). Those who did not think that the PHC should play a larger role in responding to the pandemic (n = 30, 15%) also advanced several reasons, including that the PHC did not have space to isolate patients (n = 17, 59%); was understaffed (n = 16, 53%) and underequipped (n = 12, 40%); and that reaching out might negatively affect capacity to provide other more essential services (n = 15, 50%). These staff members also suggested that instead of expansion in COVID-19 roles the PHC should focus more on provision of medications (n = 16, 57%) and delivery of immunizations (n = 14, 50%).

## Discussion

This is the first study attempting a comprehensive analysis of the impact that COVID-19 had on the Lebanese PHCN in 2020, at the beginning of the pandemic, from the perspective of both the service users and service providers. This time period also coincided with a series of compounding crises affecting the economy and social stability of the country, factors which might have contributed to the documented changes in utilization and provision of services in ways that are difficult to discriminate and measure precisely. Further, this early time frame in the pandemic corresponded to a period in which COVID-19 protocols and standard operating procedures were still being defined and the full potential role of the PHCN had not yet been acknowledged or realized [20].

We offer a summary of our findings, as well as a comparison with what has been documented so far in Lebanon and globally. Our intent is that all actors intervening in support of the Lebanese public health system adopt measures to capitalize on the strengths and mitigate the weaknesses we have identified.

### Summary of findings

In the first months after the onset of the COVID-19 pandemic in Lebanon, there was a continuity in utilization of PHC services from the patients’ side, as well as a continuity in the offer of essential services from the PHCN. The services that were most negatively affected during that period were those related to immunization and NCDs. However, it seems that the disruption was provoked mainly by economic barriers rather than by the implementation of lockdown measures. This finding might explain why the Lebanese population, rendered increasingly vulnerable from multidimensional poverty, turned to the public network to access essential health care services.

The perceived quality of services offered by the health workforce and received by the patients was seen as acceptable with regards to the implementation of IPC measures. However, patients attending the PHCN reported receiving less health information and communication than what was reported delivered by the staff.

Both patients and the health workforce considered that the PHCN could–and should–have played a larger role in the country’s response to the pandemic.

As a new epidemic is today hitting the country, with cholera spreading across the most vulnerable communities, starting once again in the poorest areas in Akkar and the North, the findings from this study might be of critical relevance in informing the ongoing response to the multifaceted humanitarian crisis now unfolding in Lebanon [18].

### Interpretation of findings

The population participating in our study seem to confirm that the service users of the Lebanese PHCN are the most vulnerable groups: in fact, more than one third (37%) of study participants were refugees, and over half (58.1%) were unemployed. Such findings underline the importance of the PHCN as a safety net in a situation of fragility and support its efforts to ensure UHC [16]. Our findings on the geographic dimension of vulnerability in Lebanon are corroborated by other studies conducted in the country under the lead of United Nations Office for the Coordination of Humanitarian affairs [21], which identify Akkar and the North Governorates as the most vulnerable regions.

Despite 2020 being the year marking the beginning of the COVID-19 pandemic, these months saw a marginal increase in the number of patients attending the PHCN in Lebanon compared to 2019 (145 new patients). The overall services interruption experienced by patients was lower than what we had conservatively assumed based on data available from the country (i.e., 15% versus 50%) [19, 22]. The workforce of the PHCN had a perception of higher interruption of services (33%) compared to those who used the services, probably due to the increased difficulties the providers faced in trying to ensure continuity of service provision [23].

In terms of specific type of services, NCD care emerged as the most severely interrupted, as has been reported in several regions of the world [1, 24]. Immunization was also mentioned as an important forgone service. This is consistent with other studies conducted in Lebanon [19], as well as with findings from the global literature [25].

What emerged as elements supporting continuity of PHC service delivery in the PHCN were not only the greater affordability of services but also a reported increased knowledge of and trust in services available. This element of increased awareness and knowledge of services available testifies to the learning journey of the MoPH PHC Department since the beginning of the Syrian crisis and its spillover effects on Lebanon [26]. The PHCN has sought the opportunity- throughout the refugee crisis and the subsequent shocks that have put it under strain- to strengthen its capacity to respond to the needs of vulnerable populations in the country and to address what had been highlighted as key challenges for them in promoting utilization of essential health care services [17, 27].

Our study highlights how the COVID-19 pandemic, associated in time with the additional shocks affecting Lebanon from 2019 onwards, has hit particularly hard on the most disadvantaged populations in Lebanon. In fact, the vast majority of reported interruptions in the utilization of PHC services was reported by the non-Lebanese population in the country. This finding adds to the body of evidence that compounding shocks often have an inequitable distribution, as they disproportionately affect refugee communities [28, 29].

The several crises that have beset Lebanon in the last five years have had a cumulative impact on the continuity of core health services. It is difficult to unpack the effect of any single shock. Health systems are increasingly being described as complex adaptive systems [30], and in a setting affected by protracted crises, such as Lebanon, it might prove particularly relevant to adopt a resilience lens in analyzing the responses to compounding stressors [31]. Particularly in fragile and conflict-affected states, key dimensions of resilience within health systems are found in the assessment of interdependencies among relevant economic, socio-political, and epidemiological factors as well as in analysis of the overall uncertainties generated by the deepening volatility of society with associated greater vulnerability of the affected populations [32]. The intersections between epidemics and poverty have been well described in other geographical settings [33, 34], but have been poorly documented in the Middle East. More in-depth studies are warranted looking at the systems dynamics unfolding in this specific region. In particular, it would be useful to adopt methodological approaches based on systems thinking to unpack the complexity of the ecosystem within which health systems operate [35].

Interestingly, pharmacies emerge as an ancillary coping mechanism for patients who seek to access drugs and to obtain health information. According to the latest survey conducted, there are 2968 community pharmacies in Lebanon, which represents a significantly broader and more easily accessible network for patients than the PHCN, particularly in remote areas [36]. It has been well documented how, throughout the pandemic, pharmacists have often turned to be frontliners in the response, emerging as the preferential entry point into care for patients who could not afford other services [37–39]. However, there has been little engagement with them from MoPH as well as from the humanitarian sector. Our findings might be a case in point for considering integrating community pharmacies in the PHCN, as has already been advocated [40].

Patients reported good adherence of PHCs to IPC measures, particularly with regards to wearing masks (99%), physical distancing (95%), and temperature control (93%), while they reported less satisfaction with education and information received (70%). In particular, non-Lebanese patients reported lower satisfaction with education and information sessions compared to Lebanese patients. These findings appear to be confirmed also beyond COVID-19 services: another study conducted in Lebanon among NCD patients highlighted the disparity in access to services and education. When comparing the provision of health services by nationality, Syrian refugees were less likely to receive the necessary services and lacked awareness on the management of their conditions [29].

The perception of the staff with regards to the implementation of IPC measures was different than that of patients. In fact, the staff reported lower availability of PPE and slightly higher levels of awareness, education and information provided to the communities. This divergence in reports is a point that warrants further research, considering the conflicting reports in the literature with regards to adherence to IPC measures among physicians in Lebanon [41].

Despite the lack of essential diagnostics and the poor options for risk communication available, patients experienced the PHCN as more accessible and affordable than other types of services. Both patients and staff believed that the PHCN should have played a larger role in responding to the pandemic, strengthening the need for a greater role for PHCs in epidemic preparedness and response, as already advocated for in Lebanon [20].

The key role of the PHCN in terms of proximity and understanding the needs of the patients makes it the ideal candidate not only for early warning in the countryside and provision of health security at country level, but also more broadly for UHC. Despite the development of early warning systems in the Middle East, it has in fact been highlighted how their performance remains substantially substandard in terms of population coverage [42]. In the context of the fragmented Lebanese PHC system, effectiveness of epidemic surveillance is jeopardised by the weak integration of the private network into the MoPH surveillance system. It is therefore imperative to take stock of these findings particularly in this historical moment, where Lebanon is facing a cholera epidemic which is adding more strain to an already overburdened health system [18]. Investing in the PHCN now, at this pivotal time, might help decrease the burden on hospitals as well as provide multiple entry points into prevention and care for the most vulnerable, supporting the efforts of the PHCN to progress towards UHC.

### Strengths and limitations

Our study is the first cross-sectional study of its kind, exploring changes in utilization and provision of services in the PHCN in Lebanon through a representative sample of both service users and service providers. The fact that both patients and staff were included in the study population provides a unique opportunity to document perceived reasons for changes from multiple viewpoints; and to triangulate our findings with what has been documented in previous studies analysing only specific subgroups of service users and providers, or specific essential health care services.

There are, however, some limitations. In particular, the very long period covered by our inquiry (two years), might have inevitably led to an important recall bias. Further, multiple factors have been investigated simultaneously, which might add an unknowable element of confounding that could not be controlled for in the analysis. A second element that needs to be taken into account is the fact that the public PHCN represents approximately only 20% of the total number of facilities providing primary care at country level, with its 254 PHCs out of a complex and diverse landscape of over 1000 outlets across the country, established by private profit and non-for-profit actors who did not systematically provide routine health services data to the MoPH [17]. Therefore, the findings here might not be generalizable to the whole population. We consider them still relevant nevertheless, as this information is systematically drawn from the most vulnerable populations in country who are unlikely to have access to non subsidised care and are therefore more likely to be the main users of the public network. This study provides insight into the barriers to accessing primary care for those who are the most at risk of being left behind, with the added value of identifying barriers that are specific to them.

The consistency between the findings of our study and those reported earlier in the literature corroborates the validity of our results, although the study was not powered for the substantially smaller change in utilisation of services documented compared to what was conservatively expected (i.e. 15% versus 50%). There was no evidence, before we analyzed our results, pointing towards such a small change in utilisation, as the literature available indicated a much greater negative impact of COVID-19 on utilisation of essential health care services in Lebanon [19, 25]. Despite the discrepancy between what was expected and what evolved in terms of response rate, the findings themselves that emerged are consistent these other studies and corroborate the hypothesis that specific types of services were particularly negatively affected—namely immunization and NCD care [19, 29].

It is also the case that the ultimate sample used in this study was less than half of the original sample sought in our initial sampling frame. Reasons for the progressive exodus of patients and PHCs from our study are briefly mentioned (not reachable, not eligible, reluctance to participate). It is possible that there are underlying factors which may have skewed our final sample in unknown ways. To address this concern, a subsequent analysis of our initial outreach sample by age, sex, and nationality showed marked similarity in demographic parameters with the sample reached for our study.

### Implications for public health practice and research

Our study sheds light on the critical role of the PHCN in Lebanon as the last resort provider in times of hardship for the most vulnerable populations in the country. It is therefore imperative that donors and the aid community join efforts in ensuring additional support to strengthen this key contribution, particularly in this critical moment of intensifying economic crisis, collapse of essential services such as electricity and sanitation, removal of subsidies for essential drugs, and an ongoing cholera outbreak.

The PHCN has proven resilient to the cascading shocks affecting the country and has learned lessons from the past. Engagement with communities and stronger risk communication emerge as an area of further improvement that will need additional support from the international community.

The emerging role of pharmacies as frontline providers warrants further investigation, as it might be an element to consider in the development of the PHCN as it seeks to address the growing humanitarian needs of vulnerable Lebanese and refugee populations in the country.

## Conclusions

The added value of the Lebanese PHCN in the provision of essential preventive and curative health care services has recently been acknowledged [16], and its potential unlocked, only at a later stage of the unfolding of the pandemic, specifically after our data collection was carried out. Our study highlights, however, that since the beginning of the COVID-19 pandemic, the PHCN has proved resilient in ensuring an unexpectedly strong continuity of service availability for the most vulnerable populations in the country. With the ongoing cholera outbreak and the uncertainty ahead, it is essential to sustain support for this vital network, in order to avoid disruptions and to protect the health security of those many whom the international community has pledged not to leave behind.

## Supporting information

S1 File (DOCX)

## Abbreviations

CI: Confidence Interval
COVID-19: Coronavirus disease-2019
ICRC: International Committee of the Red Cross
IPC: Infection prevention and control
LMICs: Low and lower-middle income countries
MoPH: Ministry of Public Health
NCD: Non communicable disease
PHC: Primary Health care Center
PHCN: Primary Health care Network
PHENICS: Primary Health Care Network Information and Communication System
PPE: Personal protective equipment
SARS-COV-2: Severe acute respiratory syndrome coronavirus 2
SD: Standard deviation
UHC: Universal Health Coverage
WHO: World Health Organisation
