# Supplementary material for: Has the COVID-19 pandemic changed the utilization and provision of essential health care services from 2019 to 2020 in the primary health care network in Lebanon? Results from a nationwide representative cross-sectional survey
Source: PLoS One. 2023 Jul 13;18(7):e0288387. doi: 10.1371/journal.pone.0288387 (PMC10343078; doi:10.1371/journal.pone.0288387)
Supplement: S1 File — (DOCX) [file pone.0288387.s001.docx]

**SUPPLEMENTARY MATERIAL**

**Supplementary material 1.** Distribution of the sampled PHCs across Lebanon (N=96)


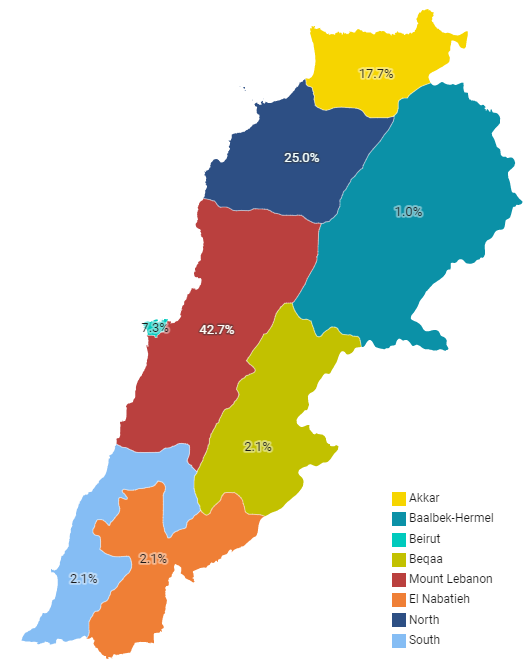


**Supplementary material 2. Characteristics of the Lebanese and non-Lebanese patients.**

| Characteristic | | Lebanese | | Non-Lebanese | | p-value |
| --- | --- | --- | --- | --- | --- | --- |
|  |  | n | Percentage (%) | n | Percentage (%) |  |
| Age (Mean, SD) | | 42.4, 13.9 |  | 35.7, 9.6 |  | <0.001 |
| Gender (N=749) | | | | | | |
|  | Male | 172 | 36.8 | 152 | 53.7 | <0.001 |
|  | Female | 295 | 63.2 | 130 | 45.9 |  |
| Beneficiary (N=754) | | | | | | |
|  | Primary beneficiary | 232 | 48.8 | 99 | 35.0 | <0.001 |
|  | Caregiver: | 243 | 51.2 | 180 | 63.6 |  |
|  | for child | 188 | 77.4 | 151 | 83.9 | 0.271 |
|  | for adult | 47 | 19.3 | 25 | 13.9 |  |
|  | unknown | 8 | 3.3 | 4 | 2.2 |  |
| Governorate (N=758) | | | | | | |
|  | Akkar | 89 | 18.7 | 45 | 15.9 | 0.149 |
|  | Baalbak El Hermel | 4 | 0.8 | 4 | 1.4 |  |
|  | Beirut | 33 | 6.9 | 23 | 8.1 |  |
|  | Bekaa | 12 | 2.5 | 4 | 1.4 |  |
|  | El Nabatieh | 10 | 2.1 | 6 | 2.1 |  |
|  | Mount Lebanon | 194 | 40.8 | 127 | 44.9 |  |
|  | North | 128 | 26.9 | 63 | 22.3 |  |
|  | South | 5 | 1.1 | 11 | 3.9 |  |
| Year of attendance to PHC (N=758) | | | | | | |
|  | 2019 only | 80 | 16.8 | 36 | 12.7 | 0.194 |
|  | 2020 only | 83 | 17.5 | 60 | 21.2 |  |
|  | 2019 and 2020 | 312 | 65.7 | 187 | 66.1 |  |
| Employment status of beneficiary (N=753) | | | | | | |
|  | Unemployed | 280 | 59.4 | 158 | 56.0 | 0.357 |
|  | Employed | 191 | 40.6 | 124 | 44.0 |  |
|  | Full-time | 94 | 49.2 | 28 | 22.6 | <0.001 |
|  | Part time | 57 | 29.8 | 43 | 34.7 |  |
|  | Seasonal | 31 | 16.2 | 41 | 33.1 |  |
|  | Self-employed | 9 | 4.7 | 12 | 9.7 |  |
| Occupation of employed beneficiary (N=315) | | | | | | |
|  | Private sector | 85 | 46.7 | 22 | 18.0 | <0.001 |
|  | Public sector | 27 | 14.8 | 5 | 4.1 |  |
|  | Daily worker | 58 | 31.9 | 93 | 76.2 |  |
|  | Other | 12 | 6.6 | 2 | 1.6 |  |
| Employment status of breadwinner (N=742) | | | | | | |
|  | Not applicable | 55 | 11.8 | 24 | 8.7 | 0.377 |
|  | Unemployed | 125 | 26.8 | 81 | 29.3 |  |
|  | Employed | 286 | 61.4 | 171 | 62.0 |  |
|  | Full-time | 151 | 52.8 | 42 | 24.6 | <0.001 |
|  | Part time | 78 | 27.3 | 59 | 34.5 |  |
|  | Seasonal | 45 | 15.7 | 55 | 32.2 |  |
|  | Other | 12 | 4.2 | 15 | 8.8 |  |
| Occupation of employed breadwinner (N=456) | | | | | | |
|  | Public sector | 38 | 13.3 | 6 | 3.5 | <0.001 |
|  | Private sector | 116 | 40.6 | 29 | 17.0 |  |
|  | Daily worker | 95 | 33.2 | 119 | 69.6 |  |
|  | Other | 34 | 11.9 | 15 | 8.8 |  |
|  | Not specified | 2 | 1.2 | 2 | 1.2 |  |
| Education of beneficiary (N=745) | | | | | | |
|  | No education | 67 | 14.4 | 76 | 27.1 | <0.001 |
|  | Primary/Elementary school | 170 | 36.6 | 150 | 53.6 |  |
|  | Secondary/High school | 113 | 24.3 | 45 | 16.1 |  |
|  | University | 115 | 24.7 | 9 | 3.2 |  |

SD: standard deviation

OR: odds ratio

CI: confidence interval

**Supplementary material 3.** The services needed in 2020 by patients who only attended the PHC in 2019 (N=29) comparing Lebanese patients (N=15) to non-Lebanese patients (N=14).

Abbreviations: ANC: Antenatal care, NCD: non-communicable diseases.

*statistically significant p-value<0.05

**Supplementary material 4**.**a.** The reasons for not seeking the needed services from PHCs in 2020 as reported by patients who only attended the PHC in 2019 (N=29).

**Supplementary material 4.b.** The reasons for not seeking the needed services from PHCs in 2020 as reported by patients who only attended the PHC in 2019 according to nationality (N=29).

|  | **Lebanese** (N=14) | | **Non-Lebanese** (N=15) | | **p-value** |
| --- | --- | --- | --- | --- | --- |
| **Reason** | **n** | **%** | **n** | **%** |  |
| Financial barriers | 8 | 57.1 | 12 | 92.3 | 0.077 |
| Geographical barrier | 4 | 26.7 | 6 | 46.2 | 0.433 |
| Lack of needed services | 3 | 21.4 | 5 | 38.5 | 0.420 |
| PHC was prioritizing urgent visits | 2 | 14.3 | 7 | 53.8 | 0.046* |
| Lockdown measures | 1 | 7.1 | 8 | 61.5 | 0.004* |
| Fear of COVID-19 | 7 | 46.7 | 8 | 66.7 | 0.441 |
| Had work | 4 | 26.7 | 3 | 21.4 | 0.741 |
| Had to take care of family | 3 | 21.4 | 5 | 38.5 | 0.420 |

*statistically significant p-value<0.05

**Supplementary material 5.** The reasons for interruption of services as reported by patients who benefitted from services in 2020 (N=92)*.

* Financial barrier (N=92); Geographic barrier (N=87); Lack of service in PHCC (N=86); PHCC prioritizing urgent visits (N=90); Lockdown measures (N=87); Fear of COVID19 (N=89); Had to work (N=88); Had to take care of family/relatives (N=87).

**Supplementary material 6.** General reasons for interruption and personal constraints/difficulties in the provision of services as reported by PHC staff.

|  | Yes | | No | | I don’t know | | N |
| --- | --- | --- | --- | --- | --- | --- | --- |
|  | n | % | n | % | n | % |  |
| General reason for interruption in provision of services | | | | | | | |
| Financial/economic issues that the staff faced (salary cuts, salary delays, insufficient salary due to Lebanese pound devaluation..) | 47 | 74.6 | 15 | 23.8 | 1 | 1.6 | 63 |
| Layoffs that took place due to the economic crisis resulting in staff shortages | 42 | 67.7 | 16 | 25.8 | 4 | 6.5 | 62 |
| Staff changing jobs or relocating out of Lebanon due to the economic crisis | 38 | 59.4 | 23 | 35.9 | 3 | 4.7 | 64 |
| Geographical access for the staff (roadblocks, distance, cost of the fuel) | 44 | 69.8 | 17 | 27.0 | 2 | 3.2 | 63 |
| Staff fear of contracting COVID-19 | 42 | 67.7 | 16 | 25.8 | 4 | 6.5 | 62 |
| Staff contracted COVID-19 | 43 | 69.4 | 18 | 29.0 | 1 | 1.6 | 62 |
| Staff affected by lockdown measures | 47 | 74.6 | 13 | 20.6 | 3 | 4.8 | 63 |
| Service hours have been reduced | 29 | 48.3 | 28 | 46.7 | 3 | 5.0 | 60 |
| Vaccines stock out | 28 | 45.2 | 33 | 53.2 | 1 | 1.6 | 62 |
| Medication stock out | 44 | 72.1 | 13 | 21.3 | 4 | 6.6 | 61 |
| Medical equipment/supplies stock out | 34 | 54.8 | 28 | 45.2 | 0 | 0.0 | 62 |
| Personal constraints/difficulties in providing services | | | | | | | |
| My financial arrangements at the PHCC were no longer satisfactory (salary cuts, salary delays, insufficient salary due to lira devaluation..) | 132 | 68.0 | 62 | 32.0 | - | - | 194 |
| I could not access the PHCC due to road blocks, distance, cost of fuel | 111 | 56.3 | 86 | 43.7 | - | - | 197 |
| Shortage of staff increased my workload/made it harder to do my job | 78 | 40.2 | 116 | 59.8 | - | - | 194 |
| I was worried I would contract COVID-19 | 137 | 70.6 | 57 | 29.4 | - | - | 194 |
| I contracted COVID-19 | 76 | 39.0 | 119 | 61.0 | - | - | 195 |
| The lockdown measures made it harder to reach/do my job | 86 | 44.8 | 106 | 55.2 | - | - | 192 |
| I felt burnt out/suffered from mental health issues | 89 | 45.6 | 106 | 54.4 | - | - | 195 |
| Vaccines stock out | 56 | 28.9 | 138 | 71.1 | - | - | 194 |
| Medication stock out | 118 | 61.1 | 75 | 38.9 | - | - | 193 |
| Medical equipment/supplies stock out | 84 | 43.3 | 110 | 56.7 | - | - | 194 |

**Supplementary material 7.** COVID-19 measures undertaken in the PHC as reported by the patients

| Measure | Yes | | No | | Total |
| --- | --- | --- | --- | --- | --- |
|  | **n** | **%** | **n** | **%** |  |
| Temperature checked at entrance | 332 | 93.0 | 25 | 7.0 | 357 |
| Questionnaire | 288 | 80.7 | 69 | 19.3 | 357 |
| Social distancing | 338 | 94.7 | 19 | 5.3 | 357 |
| Staff wearing masks | 354 | 99.2 | 3 | 0.8 | 357 |
| Available hand sanitizer | 342 | 96.3 | 13 | 3.7 | 355 |
| Patients wearing masks | 357 | 99.2 | 3 | 0.8 | 360 |
| Receive educational information | 253 | 70.5 | 106 | 29.5 | 359 |

**Supplementary material 8.** Measures taken in the PHC to protect the staff and the patients during the COVID-19 pandemic as reported by staff

| Measure | | Yes | | No | | I don't know | | Total |
| --- | --- | --- | --- | --- | --- | --- | --- | --- |
|  |  | **n** | **%** | **n** | **%** | **n** | **%** |  |
| To protect the staff | | | | | | | | |
| Training on COVID-19 triage, screening, and referral | | 119 | 60.4 | 78 | 39.6 | 0 | 0.0 | 197 |
| Training on using and removing ppe gear | | 134 | 68.4 | 62 | 31.6 | 0 | 0.0 | 196 |
| Having a separate room for symptomatic and COVID-19 suspected patients | | 127 | 64.5 | 38 | 19.3 | 32 | 16.2 | 197 |
| Availability/accessibility to sufficient quantities of: | |  |  |  |  |  |  |  |
|  | Disposable surgical masks | 175 | 88.8 | 14 | 7.1 | 8 | 4.1 | 197 |
|  | N95/FFP2 masks | 136 | 69.7 | 47 | 24.1 | 12 | 6.2 | 195 |
|  | Eye protection (goggles/face shields) | 138 | 71.1 | 41 | 21.1 | 15 | 7.7 | 194 |
|  | Examination gloves | 171 | 88.1 | 15 | 7.7 | 8 | 4.1 | 194 |
|  | Hand washing/sanitizing material for the staff | 183 | 92.9 | 7 | 3.6 | 7 | 3.6 | 197 |
|  | Cleaning material for the PHCC | 174 | 89.7 | 10 | 5.2 | 10 | 5.2 | 194 |
|  | Regular cleaning of common surfaces | 173 | 90.1 | 8 | 4.2 | 11 | 5.7 | 192 |
| To protect the patients: | | | | | | | | |
| Screening questionnaire | | 149 | 76.0 | 26 | 13.3 | 21 | 10.7 | 196 |
| Temperature check | | 187 | 96.4 | 4 | 2.1 | 3 | 1.5 | 194 |
| Triage area | | 145 | 74.7 | 37 | 19.1 | 12 | 6.2 | 194 |
| Physical distancing | | 173 | 89.6 | 17 | 8.8 | 3 | 1.6 | 193 |
| Increased time between consultations | | 154 | 79.0 | 32 | 16.4 | 9 | 4.6 | 195 |
| Limiting the number of individuals (other than staff) inside the phc | | 165 | 85.1 | 17 | 8.8 | 12 | 6.2 | 194 |
| Limiting the number of accompanying individuals | | 176 | 91.2 | 11 | 5.7 | 6 | 3.1 | 193 |
| Staff using ppe | | 177 | 92.7 | 7 | 3.7 | 7 | 3.7 | 191 |
| Patients wearing masks | | 178 | 92.2 | 8 | 4.1 | 7 | 3.6 | 193 |
| Availability of disinfectant at the entrance | | 171 | 89.5 | 9 | 4.7 | 11 | 5.8 | 191 |
| Availability of masks for symptomatic and covid-19 suspected patients | | 156 | 81.7 | 23 | 12.0 | 12 | 6.1 | 191 |
| Cleaning/disinfecting medical equipment between consultations* | | 176 | 92.1 | 6 | 3.1 | 9 | 4.7 | 191 |
| To protect the community: | |  |  |  |  |  |  |  |
| Channel for dissemination of COVID-19 awareness messages | |  |  |  |  |  |  |  |
|  | Availability of IEC in the PHC | 133 | 94.3 | 5 | 3.5 | 3 | 2.1 | 141 |
|  | Distribution of IEC to the community | 106 | 81.5 | 18 | 13.8 | 6 | 4.6 | 130 |
|  | Social media messaging | 104 | 74.8 | 20 | 14.4 | 15 | 10.8 | 139 |
|  | Home visits | 43 | 30.1 | 74 | 51.7 | 26 | 18.2 | 143 |
|  | Disseminating messages through municipalities | 102 | 71.8 | 27 | 19.0 | 13 | 9.2 | 142 |
| Methods to deal with rumors and misinformation in the community | |  |  |  |  |  |  |  |
|  | Answered patient concerns in the PHC | 167 | 86.5 | 9 | 4.7 | 17 | 8.8 | 193 |
|  | Spread awareness messages through different channels | 146 | 75.6 | 19 | 9.8 | 28 | 14.5 | 193 |
|  | Followed-up with confirmed COVID-19 cases and provided patient education | 144 | 74.2 | 18 | 9.3 | 32 | 16.5 | 194 |

IEC: information, education and communication

* Methods to decontaminate medical equipment were: disinfection (n=137, 71%), sterilization (n=136, 71%) and cleaning (n=136, 71%)

**Supplementary material 9.** Improvements needed for PHC services according to patients.

| Improvement area | Yes | | No | | Total |
| --- | --- | --- | --- | --- | --- |
|  | **n** | **%** | **n** | **%** |  |
| Availability of free medications | 689 | 91.0 | 68 | 9.0 | 757 |
| Availability of a variety of medications | 674 | 89.9 | 76 | 10.1 | 750 |
| Having more consultation time | 514 | 68.6 | 235 | 31.4 | 749 |
| Having less waiting time | 543 | 71.9 | 212 | 28.1 | 755 |
| Improving staff attitude | 257 | 34.1 | 496 | 65.9 | 753 |
| Increasing the number of staff* | 316 | 42.1 | 435 | 57.9 | 751 |
| Increasing the number of specialties* | 573 | 76.5 | 176 | 23.5 | 749 |
| Availability of diagnostic services* | 574 | 76.9 | 172 | 23.1 | 746 |
| Availability of COVID-19 testing and management | 578 | 77.5 | 168 | 22.5 | 746 |

*A higher proportion of Lebanese patients reported the need to increase the number of staff (45.8%, p-value=0.007), increase the number of specialties (81.2%, p-value<0.001), and availability of diagnostic services (79.9%, p-value=0.014) as areas for improvement compared to non-Lebanese patients (35.7%, 68.2%, and 72.2%, respectively).
